# Significance of EpCAM and TROP2 expression in non-small cell lung cancer

**DOI:** 10.1186/1477-7819-10-53

**Published:** 2012-04-06

**Authors:** Min Gyoung Pak, Dong Hoon Shin, Chang Hun Lee, Min Ki Lee

**Affiliations:** 1 Department of Pathology, School of Medicine, Pusan National University, Beomeo-ri, Mulgeum-eup, Yangsan 626-770, South Korea; 2 Department of Internal Medicine, Pusan National University, Yangsan 626-770, South Korea; 3 Medical Research Institute, Pusan National University, Yangsan 626-770, South Korea

**Keywords:** Non-small cell lung cancer, prognosis, EpCAM, TROP2

## Abstract

**Background:**

The tumor-associated calcium signal transducer (*TACSTD*) genes, originally designated epithelial cell adhesion molecule (EpCAM) and TROP2, represent true oncogenes. Little is known about EpCAM and TROP2 gene expression in non-small cell lung carcinoma (NSCLC). This study evaluated EpCAM and TROP2 protein expression and clinicopathologic significance in cases of NSCLC.

**Methods:**

Tissue microarray blocks acquired from 164 cases of NSCLC, including 100 cases of adenocarcinoma (AdC) and 64 of squamous cell carcinoma (SCC), were examined by immunohistochemical staining for EpCAM, and TROP2. The results were correlated with clinicopathologic data.

**Results:**

EpCAM and TROP2 were significantly overexpressed in SCC than in AdC (*P *< 0.01). In AdC, EpCAM overexpression was closely related to sex, histologic grade, pathologic T stage, pathologic N stage, and TNM stage, and TROP2 overexpression was only related to histologic grade (*P *< 0.05, respectively). In SCC, correlations were evident between EpCAM overexpression and TNM stage (*P *= 0.01), and between TROP2 overexpression and pathologic T stage (*P *= 0.02). EpCAM overexpression showed no significance with overall survival in AdC and SCC patients. However, TROP2 overexpression in AdC had a positive influence on overall survival (*P *= 0.02) and disease-free survival (*P *= 0.03). In particular, AdC patients with stage II or III showed better overall survival (*P *= 0.05) and disease-free survival (*P *= 0.04).

**Conclusions:**

While EpCAM and TROP2 show weak and non-complete membranous staining in normal bronchial epithelium and pneumocyte, their complete membranous expression in carcinoma suggests their role in carcinogenesis. EpCAM and TROP2 were more frequently overexpressed in SCC. EpCAM overexpression had no prognostic value in this study, but TROP2 overexpression showed better survival in AdC patients and might be a better prognostic marker in advanced stage AdC.

## Background

Non-small cell lung carcinoma (NSCLC) is a major cause of cancer-related death in bothmen and women globally [1]. Despite recent advancements in early tumor detection, surgical treatment, radiochemotherapy, and targeted therapy, the NSCLC-related high mortality rate remains a daunting challenge [2]. For example, targeted NSCLC therapy, especially against epidermal growth factor receptor (EGFR), has advanced greatly, yet only approximately 15% of NSCLCs experience therapeutic benefits due to various factors [3]. Since no single marker is sufficient for prediction of prognosis, many studies have focused on the development of new biomarkers.

The tumor-associated calcium signal transducer (*TACSTD*) gene family consists of two highly conserved and closely related genes, *TACSTD1 *and *TACSTD2*, which map to chromosomes 2p21[4] and 1p32[5], respectively. The *TACSTD1 *gene encodes TROP1, which was originally designated as epithelial cell adhesion molecule (EpCAM). TROP1 is a 39-42 kDa, 314 amino acid, type l transmembrane glycoprotein. EpCAM mediates epithelium-specific, Ca^2+^-independent homotypic cell-cell adhesion and represents the first human tumor associated antigen to be discovered. It has been used as an immunotherapeutic target of carcinoma. Normally, EpCAM is expressed on the basolateral surface of normal simple, pseudostratified, and transitional epithelia. It is also overexpressed in various human carcinomas, including those of the colon and rectum, prostate, liver, esophagus, lung, head and neck, pancreas, and breast [6]. EpCAM participates as a cell adhesion and cell signaling molecule, and its overexpression negatively correlates with survival rates in gallbladder cancer, ampullary carcinoma, and squamous cell carcinoma (SCC) of the head and neck [4,7]. EpCAM expression is also associated with better survival in clear cell renal cell carcinoma patients [8] and is related to shortened survival in node-positive breast cancer patients [9].

TROP2 is a 35 kDa, 323 amino acid, type 1 transmembrane protein with a single transmembrane domain [10]. TROP2, which was originally identified in human trophoblast and choriocarcinoma cell lines [11], is encoded by *TACSTD2*. TROP2 is not expressed in normal tissue, but is overexpressed in many carcinomas, including colorectal cancer, gastric cancer, SCC of the oral cavity, and pancreatic cancer [5]. Like EpCAM, TROP2 has been an attractive immunotherapeutic target in cancer treatment. Recently, TROP2 has been actively studied as a prognostic marker for various cancers. TROP2 overexpression has been associated with poor prognosis in colon cancer [10] and oral SCC [11].

Although the roles of EpCAM and TROP2 are not yet fully understood, both proteins are thought to participate in growth and proliferation of carcinoma cells. EpCAM can transduce an intracellular signal through its cleavage of an intracytoplasmic portion [7]. TROP2 is also believed to be a true oncogene involved in initiating signaling mechanisms that can result in increased tumorigenicity, aggressiveness, and metastasis [5]

The present study focused on the differential expression of EpCAM and TROP2 according to SCC or adenocarcinoma (AdC) histology, because certain genetic lesions may play different biological roles depending on the histological subtypes [12]. The study evaluated EpCAM and TROP2 expression in regard to various clinicopathologic factors and survival to investigate their potentials as biomarkers, and also analyzed the prognostic significance of the status.

## Methods

### Clinicopathologic data of NSCLC patients

All NSCLC patients who underwent lobectomy or pneumonectomy at Pusan National University Hospital, Busan, Korea from July 2000 to December 2009 were enrolled in this study. After exclusion of cases in which there were insufficient pathological materials remaining for further study, a total of 164 cases (113 men and 51 women; mean age 63.4 years; range 42-81 years) were selected. The patients were followed up from the date of surgery until death or the last visit to the outpatient department (median follow-up 39.4 months; range 1-123 months). At the time of the last follow-up, 129 patients (78.7%) were alive and 35 patients (21.3%) had died. Tumors were staged according to the TNM classification of the International Union Against Cancer staging system after review of the clinical, radiological, and pathological data. Other clinical information was extracted from medical records and further clincopathologic characteristics of the patients are summarized in Tables 1 and 2. There were 64 cases of SCC and 100 cases of AdC.

**Table 1:** EpCAM and Trop2 overexpression in relation to clinicopathologic factors in adenocarcinoma

Parameter	Number	EpCAM overexpression	*P *value	Trop2 overexpression	*P *value
Sex					
Male	52	26 (50.0)	0.019	13 (25.0)	0.621
Female	48	13 (27.1)		10 (20.8)	

Age at diagnosis	100		0.490		0.102

Histologic grade					
Well differentiated	42	7 (16.7)	0.000	5 (11.9)	0.015
Moderately differentiated	38	15 (39.5)		9 (23.7)	
Poorly differentiated	20	17 (85.0)		9 (45.0)	

T stage					
pT1	68	21 (30.9)	0.015	21 (24.7)	0.335
pT2, 3, 4	32	18 (56.3)		2 (13.3)	

N stage					
pN0	80	27 (33.8)	0.031	17 (21.3)	0.406
pN1, 2, 3	20	12 (60.0)		6 (30.0)	

TNM stage					
I	68	21 (30.9)	0.050	15 (22.1)	0.70
II, III	32	18 (56.3)		8 (25.0)	

Smoking	81				
Never	39	11 (28.2)	0.085	7 (17.9)	0.947
Quit	19	7 (36.8)		4 (21.1)	
Current	23	13 (56.5)		4 (17.4)	

Smoking (PY)	79		0.376		0.441

**Table 2:** EpCAM and Trop2 overexpression in relation to clinicopathologic factors in squamous cell carcinoma

Parameter	Number	EpCAM overexpression	*P *value	Trop2 overexpression	*P *value
Sex					
Male	61	52 (85.2)	0.473	40 (65.6)	0.256
Female	3	3 (100.0)		1 (33.3)	

Age at diagnosis	64		0.095		0.310

Histologic grade					
Well differentiated	7	6 (85.7)	0.815	6 (85.7)	0.240
Moderately differentiated	41	36 (87.8)		27 (65.9)	
Poorly differentiated	16	13 (81.3)		8 (50.0)	

T stage					
pT1	56	49 (87.5)	0.341	33 (58.9)	0.024
pT2, 3, 4	8	6 (75.0)		8 (100.0)	

N stage					
pN0	39	32 (82.1)	0.264	26 (66.7)	0.588
pN1, 2, 3	25	23 (92.0)		15 (60.0)	

TNM stage					
I	29	26 (89.7)	0.494	19 (65.5)	0.963
II, III	35	29 (82.9)		22 (62.9)	

Smoking	55				
Never	7	5 (71.4)	0.303	6 (85.7)	0.362
Quit	18	17 (94.4)		10 (55.6)	
Current	30	25 (83.3)		18 (60.0)	

Smoking (PY)	55		0.182		0.425

### Immunohistochemistry

Sections from TMA blocks were transferred to poly-L-lysine-coated glass slides. They were dewaxed in xylene, rehydrated in ethanol. Endogenous peroxidase activity was inactivated with 5% hydrogen peroxide in methanol for 15 min at room temperature. Antigen retrieval was performed using a cooker for 40 min at 95°C in citrate for mouse monoclonal anti-EpCAM antibody (ESA, clone VU-1D9; Novocastra, Newcastle upon Tyne, UK). Pronase treatment was carried out for purified goat polyclonal antibody against the recombinant human TROP2 extracellular domain (AF650; R&D Systems, Minneapolis, MN, USA). Antibody was incubated with the tissue sections at room temperature for 1 h. The dilutions used were 1:400 for EpCAM, and 1:50 for TROP2. For the detection of EpCAM antibody signals, an Envision detection kit (DakoCytomation, Glostrup, Denmark) was used. Labeling for TROP2 was done with ABC reagent (Vectastain Elite ABC Kit Standard PK-6100; Vector Labs, Burlingame, CA, USA). Reaction products were visualized by exposing sections to diaminobenzidine. Nuclei were lightly counterstained with Mayer's hematoxylin.

### Immunohistochemistry scoring

TMA slides were evaluated by two independent observers using light microscopy in a blinded fashion. Discordant cases were re-evaluated on a multiheaded microscope to achieve a consensus. For EpCAM, only complete membranous staining was considered for scoring. For each tumor sample, staining intensity (0, 1+, 2+, 3+) and percentage of positive tumor cells were estimated. Results were grouped as follows: negative (total absence of staining), weak (1+ staining in < 60% of cells or 2+ staining in < 30% of cells), moderate (1+ staining in ≥ 60% of cells, 2+ staining in 30% to 70%, or 3+ staining in < 30%), and strong (2+ staining in > 70% or 3+ staining in ≥ 30%) [6]. EpCAM overexpression was defined as moderate and strong immunoactivity. TROP2 expression was defined as the presence of specific staining on the surface membrane of tumor cells. TROP2 expression was evaluated for each tissue sample by calculating a total immunostaining score as the product of a proportion and intensity score. The proportion score described the estimated fraction of positively stained tumor cells (0 = none; 1 ≤ 10%; 2 = 10-50%; 3 = 51-80%; 4 ≥ 80%). The intensity score represented the estimated staining intensity (0, no staining; 1, weak; 2, moderate; 3, strong). The total score ranged from 0 to 12. TROP2 overexpression was arbitrarily defined as a total score > 4 [11].

### Statistical analyses

Pearson Chi-square test or Fisher's exact test was used to evaluate the statistical significance of immunohistochemical results related to clinicopathological parameters. Mann-Whitney test was used for continuous variables. Follow-up information was also obtained for survival analysis. Patient survival was calculated as the time between operation and death. Patients who were still alive at the time of data collection were censored in the statistical analyses. The cases lost to follow-up or deaths from any other cause were defined as censored data for the analysis of survival rates. The survival curves were plotted using the Kaplan-Meier method, and *P *values were calculated using the log-rank test. All statistical analyses were performed on a personal computer with the SPSS version 15.0 statistical package (SPSS, Chicago, IL, USA). A *P *value < 0.05 was regarded as statistically significant.

## Results

### EpCAM and TROP2 expression in normal bronchial epithelium

**Figure 1:**
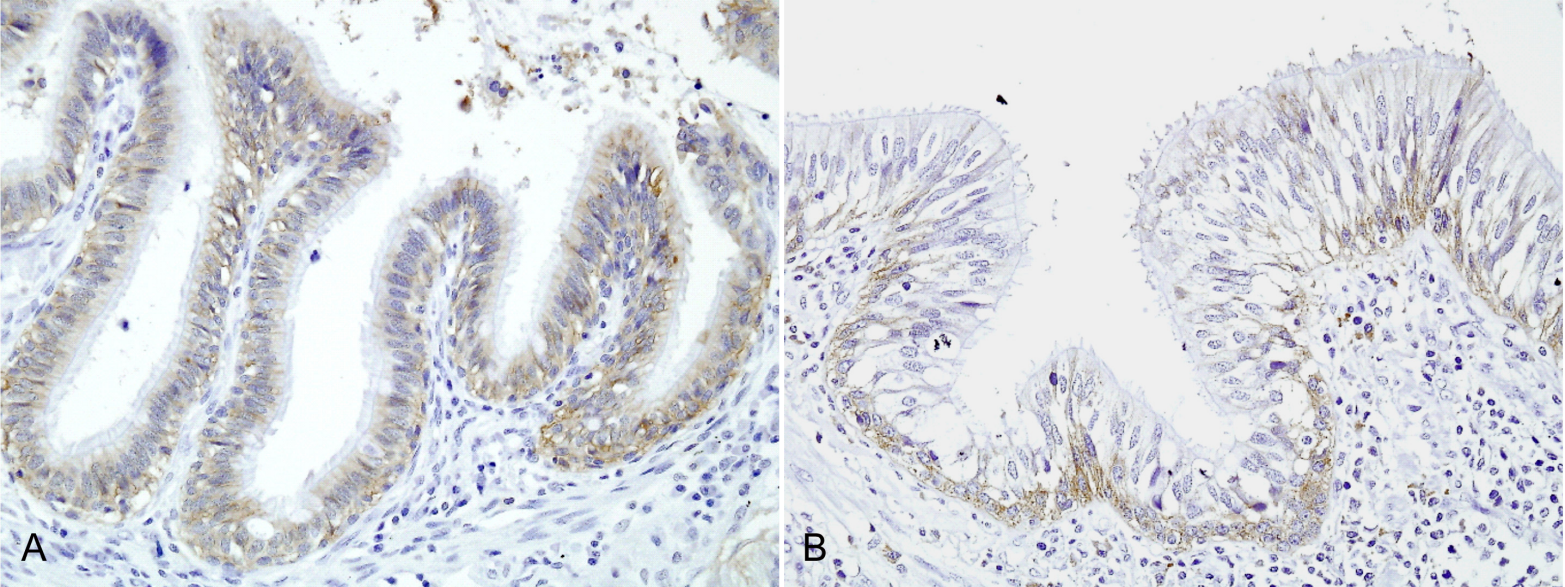
**Immunohistochemical expression of EpCAM and TROP2 in normal bronchial mucosa**. (**A**) EpCAM is expressed on the basolateral surface. (**B**): TROP2 shows a similar expression pattern.

Immunoreactivity of EpCAM and TROP2 was evaluated in 164 patents with NSCLC. EpCAM was expressed at the basolateral surface of nonneoplastic bronchial mucosa and TROP2 was absent or infrequently expressed in this location (Figure 1). Neither protein was expressed in pneumocytes.

### EpCAM expression in NSCLC

**Figure 2:**
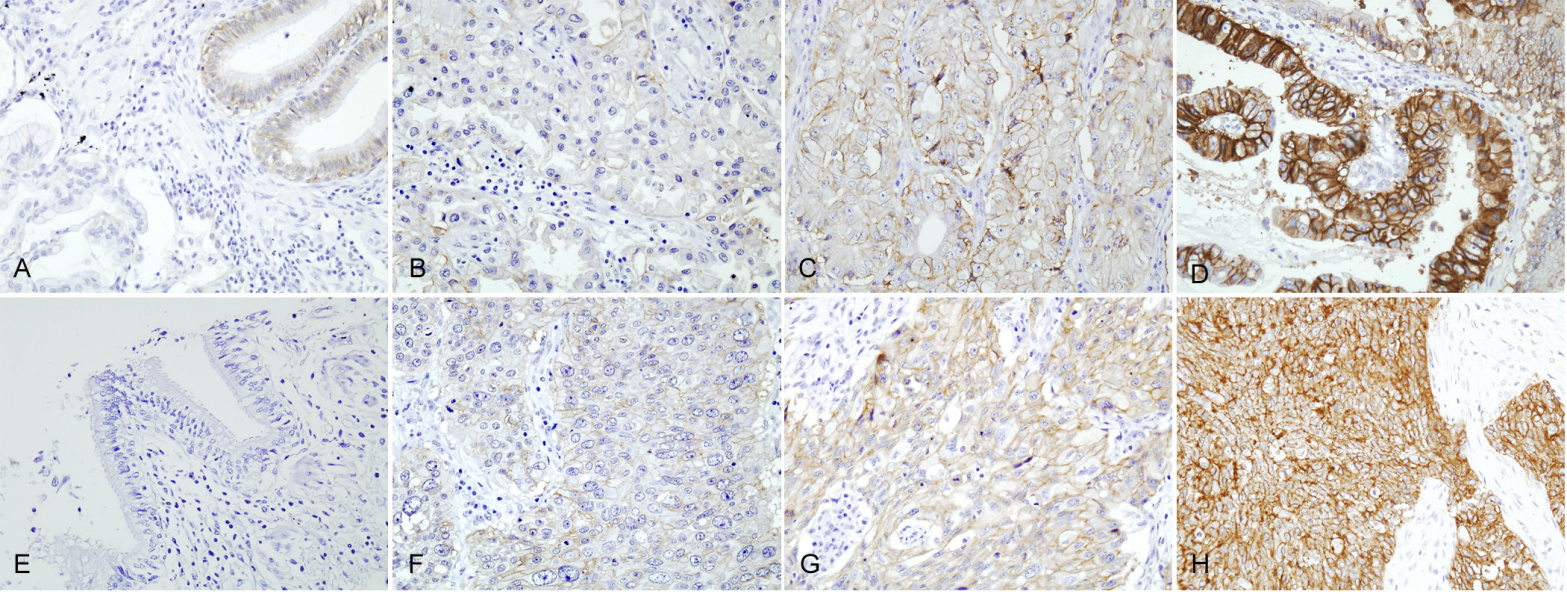
**Immunohistochemical staining for EpCAM**. Note the membranous staining of tumor cells. EpCAM expression in adenocarcinoma, without expression (**A**) and with weak (**B**), moderate (**C**), and intense (**D**) expression. EpCAM expression in squamous cell carcinoma, without expression (**E**), with weak (**F**), moderate (**G**), and intense (**H**) expression.

EpCAM showed complete membranous expression in carcinoma. From 100 AdC samples, 45 (45.0%) showed no immunoreactivity, 16 (16.0%) showed weak expression, 17 (17.0%) showed moderate expression, and 22 (22.0%) showed strong expression, accounting for an overexpression rate of 39 (39.0%) (Figure 2). From 64 SCC samples, six (9.3%) showed weak expression, 10 (15.6%) showed moderate expression, 45 (70.3%) showed strong expression, and only three (4.7%) showed no immunoreactivity, accounting for an overexpression rate of 55 (85.9%) (Figure 2). EpCAM was more frequently overexpressed in SCC than AdC (*P *< 0.01) (Table 3).

**Table 3:** EpCAM and TROP2 overexpression according to adenocarcinoma or squamous cell carcinoma

	EpCAM expression			TROP2 expression		
	
	Low	Over	P value	Low	Over	P value
Adenocaricnoma	61 (61.0)	39 (39.0)	0.000	77 (77.0)	23 (23.0)	0.000

SCC	9 (14.1)	55 (85.9)		23 (35.9)	41 (64.1)	

### TROP2 expression in NSCLC

**Figure 3:**
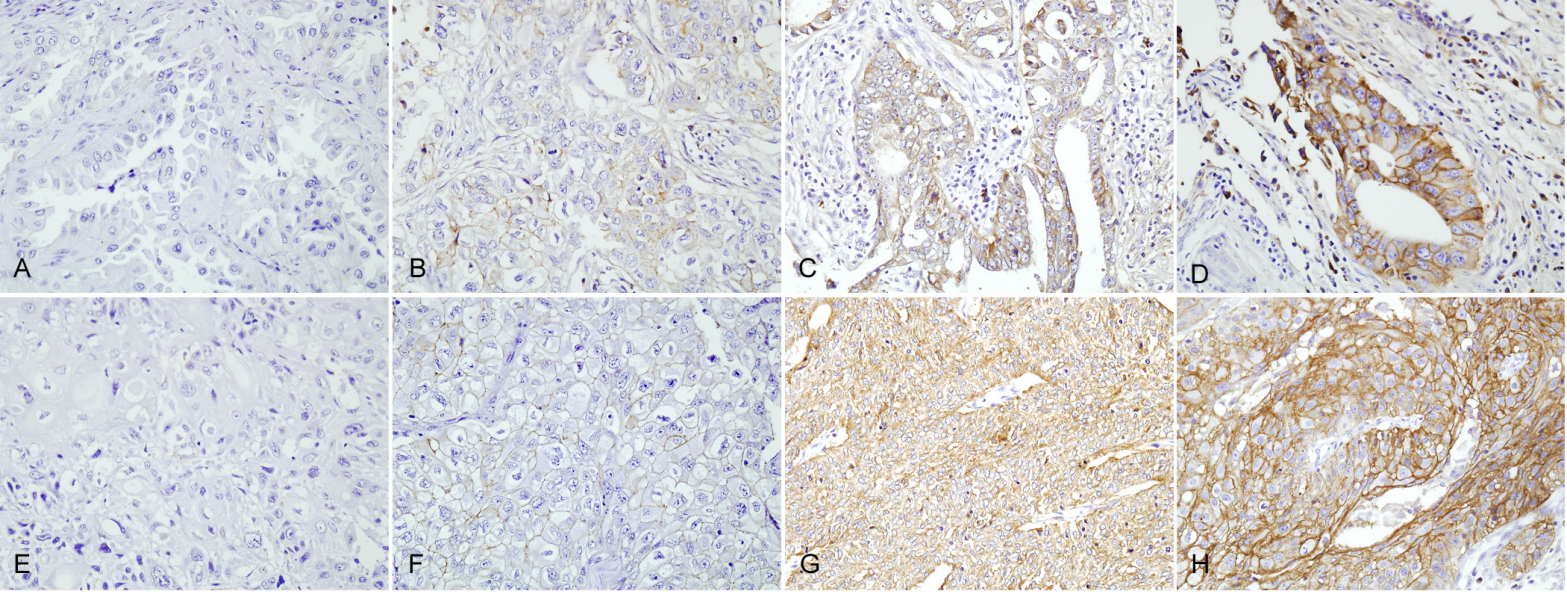
**Immunohistochemical staining for TROP2**. Note the membranous staining of tumor cells. TROP2 expression in adenocarcinoma, without expression (**A**) and with weak (**B**), moderate (**C**), and intense (**D**) expression. TROP2 expression in squamous cell carcinoma, without expression (**E**) and with weak (**F**), moderate (**G**), and intense (**H**) expression.

Immunohistochemical studies in 100 AdC samples revealed no expression (total score 0) in 53 (53.0%), weak expression (total score 1-4) in 24 (24.0%), moderate expression (total score 6 and 8) in 14 (14.0%), and strong expression (total score 9 and 12) in nine (9.0%). Thus, according to the aforementioned criteria, TROP2 was overexpressed in 23 (23.0%) of the AdC cases (Figure 3). In the 64 SCC cases, seven (10.9%) showed a lack of expression, 16 (40.6%) showed weak expression, 15 (23.4%) showed moderate expression, and 26 (32.9%) showed strong expression. TROP2 overexpression was noted in 41 (64.1%) of the SCC cases (Figure 3). TROP2 overexpression was significantly more frequent in SCC (*P *< 0.01) (Table 3).

### EpCAM and TROP2 overexpression and their correlation with clinicopathologic factors

Univariate analysis of EpCAM overexpression in relation to sex, age at diagnosis, tumor differentiation, pathologic T stage, lymph node metastasis, TNM stage, and smoking status revealed that in AdC, EpCAM overexpression was significantly associated with sex, tumor differentiation, pathologic T stage, lymph node metastatis, and TNM stage. In contrast, EpCAM overexpression was not significantly related to age at diagnosis and smoking status (Table 1). TROP2 overexpression was only associated with tumor differentiation. There was no statistical significance between TROP2 overexpression and sex, age at diagnosis, pathologic T stage, lymph node metastasis, TNM stage, and smoking status (Table 1). In SCC, EpCAM overexpression was not related to clinicopathologic factors. TROP2 showed a significant relationship only with the pathologic T stage (Table 2).

### EpCAM and TROP2 overexpression and correlation with overall survival

**Figure 4:**
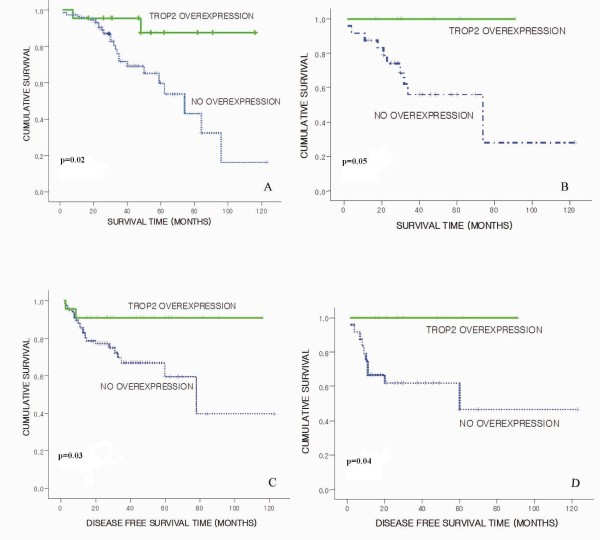
**Kaplan-Meier survival curves**. (**A**) Overall survival according to Trop2 overexpression in adenocarcinoma (AdC). (**B**) Overall survival according to according to Trop2 overexpression in stage II or III AdC. (**C**) Disease-free survival according to Trop2 overexpression in AdC. (**D**) Disease-free survival according to Trop2 overexpression in stage II or III AdC.

TROP2 overexpression showed better overall survival in AdC patients by Kaplan-Meier analysis (median follow-up 39.6 months; range 2-123 months) (*P *= 0.02) (Figure 4A). Stage II or III AdC patients with TROP2 overexpression showed better overall survival (*P *= 0.05) (Figure 4B). In SCC patients, TROP2 overexpression revealed a trend of better overall survival although it was not significant statistically (*P *= 0.49). EpCAM expression was also not related with overall survival in AdC (*P *= 0.23) or SCC (*P *= 0.61). It showed only a tendency of better survival.

### EpCAM and TROP2 overexpression and correlation with disease-free survival

TROP2 overexpression showed better disease-free survival in AdC patients (*P *= 0.03) (Figure 4C). Better disease-free survival was observed in stage II or III AdC patients with TROP2 overexpression (*P *= 0.04) (Figure 4D). In SCC patients, TROP2 overexpression is not associated with disease free survival (*P *= 0.39). However, disease-free survival was not statistically related with EpCAM expression in both histologies (AdC, *P *= 0.31; SCC, *P *= 0.60).

### Multivariate survival in AdC

In univariate survival tests with clinicopathologic factors in AdC, statistically significant were age (*P *= 0.001), T stage (*P *= 0.007), TNM stage (*P *= 0.035) besides TROP2 expression. Other variables including sex, tumor differentiation, N stage, and smoking history were not associated with better survival. Multivariate survival test was done with age, T stage TNM stage, and TROP2 expression. Only T stage (*P *= 0.033) showed significance.

## Discussion

Although the EpCAM and TROP2 proteins were discovered some time ago, only recently have they become the focus of research in various cancers. While there are increasing reports about the role of EpCAM or TROP2 in various cancers, only a few reports of EpCAM or TROP2 expression in NSCLC have been published [2,13]. In one recent study, EpCAM expression was investigated in NSCLC, but TROP2 was not involved [2]. A study by Kobayashi *et al. *has evaluated TROP2 expression in NSCLC [13], and it was limited to small-sized AdC. Hence, the present study is the first comprehensive evaluation of the expression of EpCAM and TROP2 in pulmonary AdC and SCC, simultaneously.

In the present study, we observed different patterns of EpCAM and TROP2 expression between in normal bronchial epithelium and in NSCLC. In normal bronchial epithelium, both proteins showed only infrequent basolateral cytoplasmic membrane positivity. They were not stained in alveolar pneumocytes. As EpCAM is a cell-cell adhesion molecule in epithelial cells, its distribution along the cytoplasmic membrane is not unexpected. On the other hand, EpCAM and TROP2 were overexpressed in NSCLC and their staining along the cytoplasmic membrane was complete along the membrane. This finding corroborates a previous report that the expression of EpCAM increases in a stepwise manner from uninvolved bronchial mucosa, epithelial hyperplasia, to SCC [2]. Cytoplasmic or nuclear expression was not seen in our study. Although Kobayashi *et al. *advocated that TROP2 is also expressed in cytoplasm in NSCLC [13] and we observed focal cytoplasmic staining, we think that this is non-specific staining regarding that cytoplasmic expression was only focally observed in the cases which show strong membranous TROP2 staining. Both EpCAM and TROP2 were more frequently expressed in SCC in the present study, being consistent with a previous study [6]. But, the pattern of expression was not different to each other. Besides functioning as cell-cell adhesion molecule, EpCAM has been recently known to act as an oncogene, proving to trasduce cell proliferating signal through c-myc, cyclin D, and survivin. In another aspect, the loss of EpCAM has been advocated to promote tumor progression through weakening cell-cell adhesion. When EpCAM is overexpressed, some cancers such as stage I or II gastric cancer, stage II NSCLC, clear cell renal cell carcinoma, and stage II colon cancer show better survival while others such as node-positive breast cancer, epithelial ovarian cancer, gall bladder cancer, cholangiocarcinoma, ampullary pancreas cancer, squamous cell cancer of the esophagus, and squamous cell head and neck cancer do worse. This contradicting result may be determined by which function of cell-cell adhesion or oncogene of EpCAM predominates. Although the function of TROP2 is less defined than that of EpCAM, TROP2 has also been suggested as oncogene, promoting cancer cell proliferation and migration, and cell adhesion molecule [5,14]. Regarding NSCLC, Kobayashi *et al. *reported that TROP2 overexpression in small pulmonary AdC was related with worse overall survival [13]. However, in our study, TROP2 overexpression was related with better overall survival and disease free survival in AdC, although SCC showed same tendency but no statistical significance. To our best knowledge, there has not been a study, in which TROP2 overexpression in cancer was associated with better survival than those which do not. This discordance between our study and previous one may be partially explained by that Kobayashi *et al. *included only AdC which are < 2 cm in size, while we included larger tumors of varying degree of differentiation. In our study, stage II or III AdC with TROP2 overexpression showed better overall survival and disease-free survival while in stage I AdC, TROP2 overexpression did not affect overall survival or disease-free survival. Furthermore, poorly differentiated AdCs expressed TROP2 more frequently. These data imply that poorly differentiated AdCs may show better survival when they express TROP2. Whether TROP2 serves as an adhesion molecule or not has not been fully defined. Some advocated its role as adhesion molecule [15], but early study could not prove it [16]. Recently, it has been discovered that TROP2 binds with Claudin 1 and 7 which are important components of tight junctions, taking part in cell-cell adhesion [17]. Hence, we hypothesized that the biologic role of TROP2 can be different in early and advanced pulmonary AdC. In higher stage pulmonary AdC, the role of TROP2 as an adhesion molecule may be greater than as an oncogene. TROP2 overexpression may strengthen bonds between tumor cells, preventing their shedding and progression. Like EpCAM showed variable results in variable cancers, TROP2 may play different roles in different cancers.

## Conclusions

The EpCAM and TROP2 proteins are expressed in NSCLC, and only weakly or not expressed in normal lung tissue. The expression pattern of EpCAM and TROP2 was similar in SCC and AdC. TROP2 protein did confer a significant survival effect in AdC. This finding may reflect that TROP2 is involved in cell-cell adhesion in pulmonary AdC and its loss will promote tumor cell shedding. Hence, our study showed that TROP2 predicts better prognosis in AdC of NSCLC.

## Abbreviations

NSCLC: Non-small cell carcinoma; SCC: Squamous cell carcinoma; AdC: Adenocarcinoma.

## Consent

Written informed consent was obtained from the patient for publication of this report and any accompanying images.

## Competing interests

The authors declare that they have no competing interests.

## Authors' contributions

DHS and CHL conceived the study. MGP and DHS performed the staining and interpretation. MKL collected the clinical data. MGP performed the literature review and wrote the manuscript. DHS and CHL supervised the experiments and manuscript writing. All authors read and approved the final manuscript.
